# Temporal changes of CT findings between non-severe and severe cases of COVID-19 pneumonia: a multi-center, retrospective, longitudinal Study

**DOI:** 10.7150/ijms.51159

**Published:** 2020-09-21

**Authors:** Meng Dai, Xiaoming Liu, Xiqi Zhu, Tiejun Liu, Cihao Xu, Fang Ye, Lian Yang, Yu Zhang

**Affiliations:** 1 Department of Radiology, Union Hospital, Tongji Medical College, Huazhong University of Science and Technology, Wuhan, Hubei, China.; 2 Hubei Province Key Laboratory of Molecular Imaging, Wuhan, Hubei, China.; 3 Department of Radiology, Nanxishan Hospital, Guangxi Zhuang Autonomous Region, No. 46 Chongxin Road, Guilin 541002 Guangxi, People's Republic of China.; 4 Department of Radiology, Liuzhou People's Hospital, No. 8, Wenchang Road, Liuzhou 545006, Guangxi, People's Republic of China.; 5 Department of Occupational and Environmental Health and Ministry of Education Key Lab for Environment and Health, School of Public Health, Tongji Medical College, Huazhong University of Science and Technology, Wuhan, Hubei, China.

**Keywords:** COVID-19, CT score, Ground-glass opacity, CT abnormalities, non-severe, severe

## Abstract

**Background and aim:** To perform a longitudinal analysis of serial CT findings over time in patients with COVID-19 pneumonia.

**Methods:** From February 5 to March 8, 2020, 73 patients (male to female, ratio of 43:30; mean age, 51 years) with COVID-19 pneumonia were retrospectively enrolled and followed up until discharge from three institutions in China. The patients were divided into the severe and non-severe groups according to treatment option. The patterns and distribution of lung abnormalities, total CT scores, single ground-glass opacity (GGO) CT scores, single consolidation CT scores, single reticular CT scores and the amounts of zones involved were reviewed by 2 radiologists. These features were analyzed for temporal changes.

**Results:** In non-severe group, total CT scores (median, 9.5) and the amounts of zones involved were slowly increased and peaked in disease week 2. In the severe group, the increase was faster, with scores also peaking at 2 weeks (median, 20). In both groups, the later parameters began to decrease in week 4 (median values of 9 and 19 in the non-severe and severe groups, respectively). In the severe group, the dominant residual lung lesions were reticular (median single reticular CT score, 10) and consolidation (median single consolidation CT score, 7). In the non-severe group, the dominant residual lung lesions were GGO (median single GGO CT score, 7) and reticular (median single reticular CT score, 4). In both non-severe and severe groups, the GGO pattern was dominant in week 1, with a higher proportion in the severe group compared with the non-severe group (72% vs. 65%). The consolidation pattern peaked in week 2, with 9 (32%) and 19 (73%) in the non-severe and severe groups, respectively; the reticular pattern became dominant from week 4 (both group >40%).

**Conclusion:** The extent of CT abnormalities in the severe and non-severe groups peaked in disease week 2. The temporal changes of CT manifestations followed a specific pattern, which might indicate disease progression and recovery.

## Introduction

In late December 2019, an outbreak of Coronavirus disease-19 (COVID-19) occurred in Wuhan, Hubei Province, China 1-6. Currently, this infection has been detected all over the world. On July 14, 2020, the global number of confirmed COVID-19 cases surpassed 12,964,809 7. On February 11, 2020, a novel coronavirus was identified by the Chinese Center for Disease Control and Prevention (CDC) from Chinese patients with pneumonia 2, which was named severe acute respiratory syndrome coronavirus 2 (SARS-CoV-2) by WHO 8, which formed a clade within the subgenus sarbecovirus in the orthocoronavirinae subfamily 2.

Patients with SARS-CoV-2 infection typically present with fever, cough and dyspnea 5,6. Most patients with COVID-19 have mild symptoms, whereas a few develop severe pneumonia, pulmonary edema, acute respiratory distress syndrome (ARDS), and/or multiple organ failure (MOF), or even die, similar to individuals with severe acute respiratory syndrome (SARS) and Middle East respiratory syndrome (MERS) 4, 9, 10.

Chest CT examination plays an important role in the diagnosis and management of COVID-19 pneumonia 11-16. Reports also indicated that patients with highly suspected SARS-CoV-2 infection have negative results in the initial real time reverse transcription-polymerase chain-reaction (RT-PCR) test, but positive findings on CT images 17-19.

Previous studies demonstrated CT findings to be diverse 20, 21; however, CT abnormalities between non-severe and severe COVID-19 pneumonia cases have not been well investigated. A better understanding of the progression of CT findings in COVID-19 pneumonia may help achieve accurate diagnosis and disease staging. Thus, this longitudinal study was performed to analyze serial CT findings between non-severe and severe COVID-19 pneumonia cases.

## Materials and Methods

### Patients

This retrospective study was approved by our Institutional Ethics Committee. Patient data were collected from 3 hospitals in China from Feb 5 to Mar 8, 2020. A total of 73 patients with definite COVID-19 were enrolled, including 43 males and 30 females, aged 26 to 85 years (51 ± 13 years). The diagnosis of COVID-19 was made in accordance with the Guidelines for the Diagnosis, Discharge and Treatment Criteria of New Coronavirus Pneumonia (sixth edition) 22 formulated by the National Health Commission of the People's Republic of China.

Inclusion criteria were: (1) at least two CT examinations; (2) discharged from one of the 3 hospitals by the censored date (Mar 08, 2020); (3) 1.5-2.0 mm thickness thin-section images, no artifacts affecting diagnosis. Exclusion criteria were: (1) only one CT examination, (2) severe image artifacts due to poor respiratory cooperation.

Discharge Criteria were specified as: (1) absence of fever for at least 3 days; (2) substantial improvement in both lungs upon chest CT, clinical remission of respiratory symptoms; (3) two negative results of throat-swab tests for SARS-CoV-2 RNA at an interval of at least 24 h.

CT scans were categorized according to the time duration between symptom onset and CT images acquisition, i.e., 1, 2, 3, 4, and over 4 weeks after symptom onset.

### Clinical typing and images grouping

Disease severity was classified into 4 categories according to the Guidelines for the Diagnosis and Treatment of New Coronavirus Pneumonia (sixth edition): (1) mild type, mild clinical symptoms and no pulmonary changes on CT images; (2) common type, fever and signs of respiratory infection, with pneumonia changes on CT images; (3) severe type, any of the items including respiratory distress (respiratory rate ≥ 30/min), finger oxygen saturation ≤ 93% in the resting state, arterial partial pressure of oxygen (PaO_2_) /oxygen concentration (FiO_2_) ≤ 300 mmHg (1 mmHg = 0.133 kPa) and critical type (respiratory failure requiring mechanical ventilation, shock or ICU admission requirement due to multiple organ failure).

To analyze the serial CT images, 73 patients were re-grouped into non-severe (mild and common type) and severe (severe and critical type) groups according to treatment criteria. Among the re-grouped patients, 47 (mean age, 49 ± 12 years) were recruited into the non-severe group, while 26 (mean age, 55 ±15 years) were enrolled in the severe group.

### CT Protocol

High-resolution CT scans were performed in all patients with commercial multi-detector CT scanners at the single inspiratory phase (uCT550, Shanghai United Imaging Healthcare, Shanghai, China; Aquilion 16-slice, TOSHIBA Medical Systems; Tochigi, Japan; NeuViz 64in, Shengyang Neusoft medical system, Hunan, China; Somatom Definition AS, Siemens, Nurnberg,Germany; Brilliance 64, Phillips Medical System, Eindhoven, the Netherlands). CT images were acquired under the following conditions: tube voltage 120 kVp; pitch, 1.075; collimator widths of 64×0.6 mm, 128×0.6 mm, 64×0.6 mm, and 40×0.55 mm. They were reconstructed based on the raw data with a matrix size of 512×512 as axial images (thickness, 1.5 or 2 mm; increment, 1.5 or 2 mm) in the transverse slice direction. All images were acquired with the lung (window width, 1,000~1,500 HU; window level, -700 HU) and mediastinal (window width, 350 HU; window level, 35~40 HU) settings.

### Chest CT Evaluation

All images were independently assessed by 2 senior radiology specialists (M.D. and Y.Z., with 7and 5 years of experience in interpreting chest CT findings, respectively) using the institutional digital database system (Vue PACS, version 11.3.5.8902, Carestream Health, Canada). The location, shape, number and size of the abnormalities on chest CT images were carefully observed and recorded. Decisions were reached by consensus in case of disagreement between the two radiologists.

**Table 1** illustrates all CT imaging features extracted from each CT scan of the study cohort 16, 23-26.

A semi-quantitative analysis was performed to quantify disease severity 11, 27. For each lung lobe, a total CT score representing the extent of lung involvement [ground-glass opacity (GGO), consolidation and reticular types 65%] was visually rated from 0 to 5: 0, none; 1, <5% lobe involvement; 2, 5%-25% lobe involvement; 3, 26%-49% lobe involvement; 4, 50%-75% lobe involvement; and 5, >75% lobe involvement. Single GGO, single consolidation, and single reticular CT scores were evaluated, respectively, according to the criteria described above.

### Statistical analysis

Illness day 0 was defined as the day of initial symptom onset. Median total, single GGO, single consolidation and single reticular CT scores were plotted as a function of time, as well as the amounts of zones involved. The temporal changes of main CT patterns, ground-glass opacity subtypes, and the distribution of lung abnormalities were also analyzed. The Kruskal Wallis rank-sum test was performed to compare CT lung quantification in different periods. The Chi-square test was applied to compare the frequency of CT patterns in different periods. *P* < 0.05 was considered statistically significant. Statistical analyses were performed with the R software (version 3.6.2, the R Foundation for Statistical Computing, Vienna, Austria).

## Results

### Patient characteristics

Totally 73 patients were enrolled in this study. They included 47 non-severe (32 males and 15 females, aged 49±12 years) and 26 were severe (11 males and 15 females, aged 55±15 years) cases. Among the non-severe cases, 68% were male, while 58% of severe cases were female. As for concurrent underlying diseases (**Table 2**), 56% of patients had different underlying diseases, among which hypertension (29%) and fatty liver (18%) were dominant. In the severe group, 61% of patients had non-pulmonary underlying diseases, versus 41% in the non-severe group. Fever (86%), cough (62%), and dyspnea (56%) were the most common symptoms. Over 70% of severe cases had cough and dyspnea, and diarrhea (26%) and anorexia (58%) cases were more abundant compared with the non-severe group. The time from initial symptom onset to positive nucleic acid RT-PCR was shorter in the severe group compared with the non-severe group, meanwhile, the time between two successive negative nucleic acid tests was more prolonged versus the non-severe group. In addition, the imaging peak time from initial symptom onset in the non-severe and severe groups occurred in the second week (**Table 2**).

### CT imaging manifestations

In the non-severe group, bilateral lower lobe involvements were more common and the involvement of the middle lobe of the right lung accounted for the lowest proportion among the 5 lung lobes. In the severe group, over 95% of patients had simultaneous involvement of 5 lung lobes within the initial 4 weeks of the disease course; this proportion was over 90% after 4 weeks.

In the non-severe group, over 85% of patients had a trend of bilateral lung involvement, while in the severe group, obvious bilateral lung involvement was observed within 1-week after disease onset.

In the non-severe and severe groups, disease site and scope were dominated by the central and peripheral and diffuse types, without predilection for sub-pleural or peri-bronchovascular regions (**Table 3**).

### CT scores

**Figure 1:**
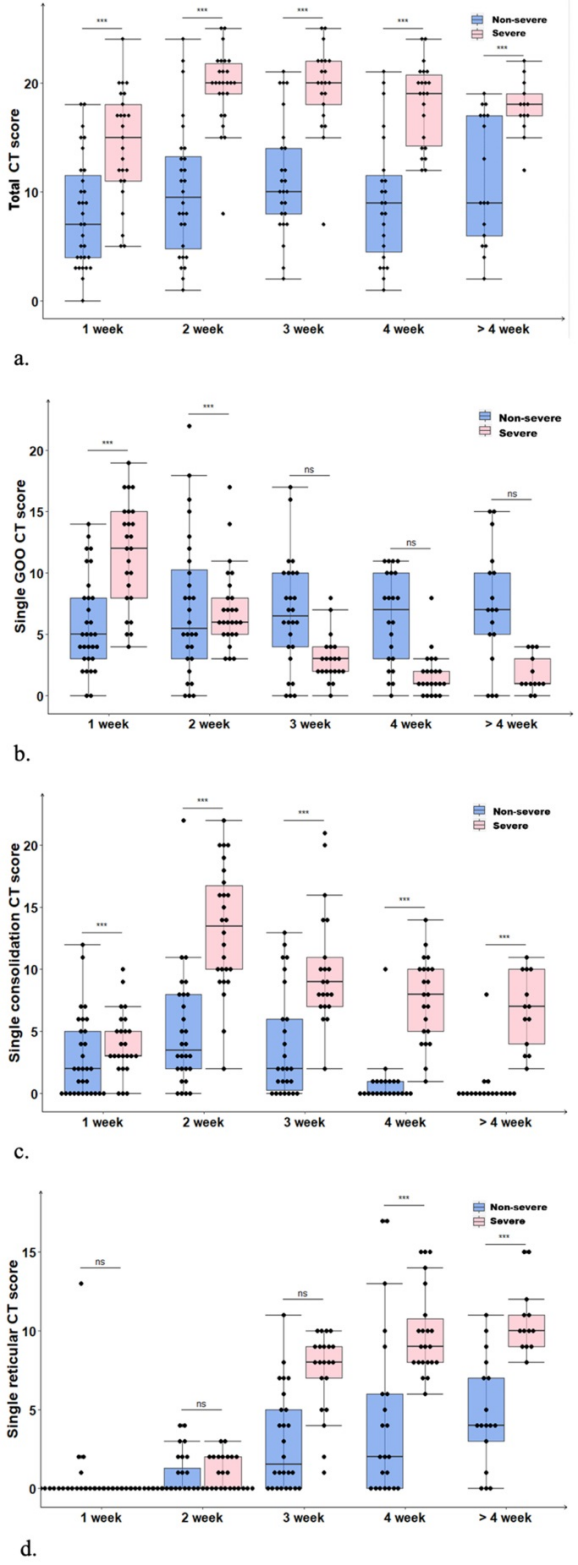
(**a**) Temporal changes of median total CT scores in the non-severe and severe groups both peaked in the 2^nd^ week, with the severe showing significantly higher values than the non-severe group (*P=*0.000). (**b**) Temporal changes of median single GGO CT scores, with the severe group showing a downward trend (*P=*0.000). (**c**) Temporal changes of median single consolidation CT scores, with the non-severe and severe groups both peaking in the 2^nd^ week, and the severe group showing significantly higher values (*P=*0.000). (**d**) Temporal changes of median single reticular CT score, with the non-severe and severe groups both peaking after 4 weeks; both the severe (*P=*0.000) and non-severe (*P=*0.000) groups showed an upward trend.

In the non-severe group, the disease slowly worsened within the first two weeks; the median total CT score was increased from 7 (range: 0-18) points in the first week to a peak of 9.5 (range: 1-24) points in the second week, but began to decline in the 4^th^ week to reach 9 (range: 2-19) points (**Fig. 1A**). Such a result revealed the presence of residual lesions at the late disease stage. Differences in total CT scores in the non-severe group at different time periods were not statistically significant (*P=*0.435). In the severe group, the disease peaked in the 2^nd^ week rapidly with a median total CT score as high as 20 (range: 8-25) points. In the 3^rd^ week, the median total CT score in the severe group remained at the peak value (20; range: 7-25 points), and it began to decline slowly in the 4^th^ week to 18 (range: 12-22) points after 4 weeks. Differences in the total CT scores in the severe group at different time periods were statistically significant (*P=*0.000). The median total CT score in the severe group at each time point was higher than the corresponding values for the non-severe group (*P=*0.000), suggesting the presence of obviously greater residual lung lesions in severe cases compared with the non-severe group.

In the non-severe group, single GGO CT scores showed no statistically significant differences among various time periods of the disease course (*P*=0.204); in the severe group, the median single GGO CT score gradually decreased from 12 (range: 4-19) points in the first week to 1 (range: 0-4) point after 4 weeks (**Fig. 1B**), indicating statistically significant differences among various time periods (*P*=0.000). In the non-severe and severe groups, median single consolidation CT scores peaked in the second week, with 4 and 14 points, respectively; the lowest scores in the above two groups were obtained after 4 weeks, i.e., 0 and 7 points, respectively (**Fig. 1C**). Differences in single consolidation CT scores at different time periods were statistically significant in the non-severe group (*P=*0.000); the median single consolidation CT score at each time point was higher in the severe group compared with the non-severe group (*P*=0.000). In the non-severe and severe groups, median single reticular CT scores were low in the first two weeks, gradually elevated from the second week, and peaked after 4 weeks (4 and 10 points in the non-severe and severe groups, respectively) (**Fig. 1D**). Differences in single reticular CT scores at different time periods in non-severe (*P*=0.000) and severe (*P*=0.000) cases were statistically significant, and scores were remarkably higher in the severe group compared with the non-severe group after 3 weeks. In severe cases, the residual lung lesions were mostly of reticular and consolidation types, with reticular ones being more common. By contrast, most residual lung lesions in the non-severe group were GGO and reticular, with GGO being more common.

### Predominant CT patterns

**Figure 2:**
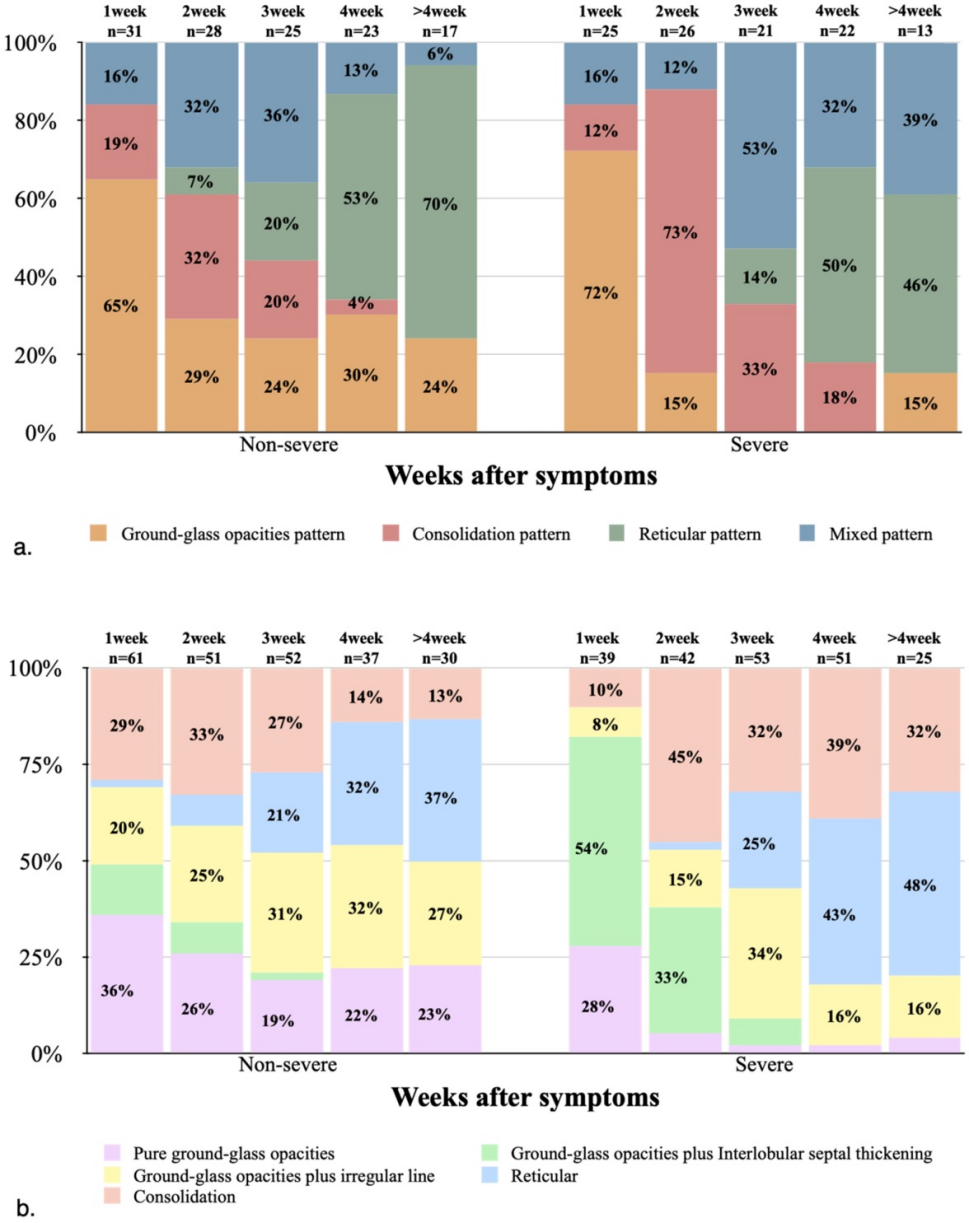
(**a**) Stacked-bar graph showing temporal changes of the predominant CT patterns in the non-severe and severe groups at various time points from the onset of symptoms. (**b**) Stacked-bar graph showing the amounts of positive CT features in the non-severe and severe groups at various time points from the onset of symptoms.

**Figure 3:**
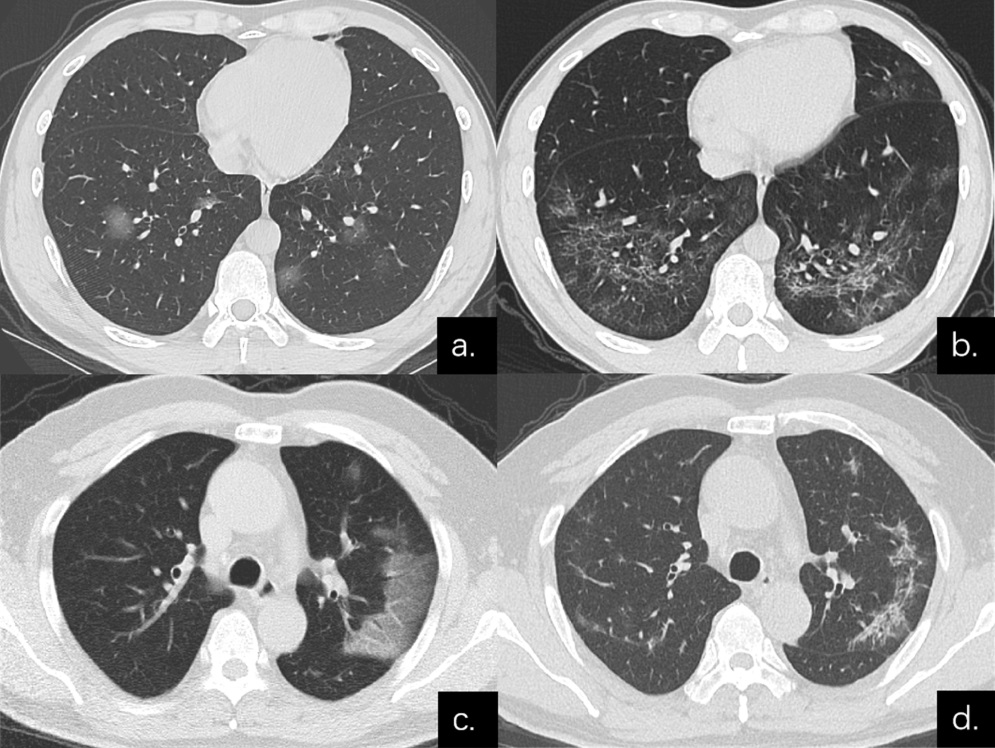
Series CT scans in a 38-year-old man with COVID-19 pneumonia in the non-severe group. (**a**) Scan obtained on disease day 6 showed a predominant GGO pattern with multiple pure GGO in the lower lobes. (**b**) Scan obtained on day 31 showed a predominant reticular pattern with interlacing line shadows that suggest a mesh mainly in the lower lobes. Series CT scans in a 54-year-old man with COVID-19 pneumonia in the non-severe group. (**c**) Scan obtained on disease day 1 showed GGO in left upper lobe; predominant GGO pattern. (**d**) Scan obtained on day 27 showed a predominant reticular pattern with interlacing line shadows mainly in left upper lobe.

**Figure 4:**
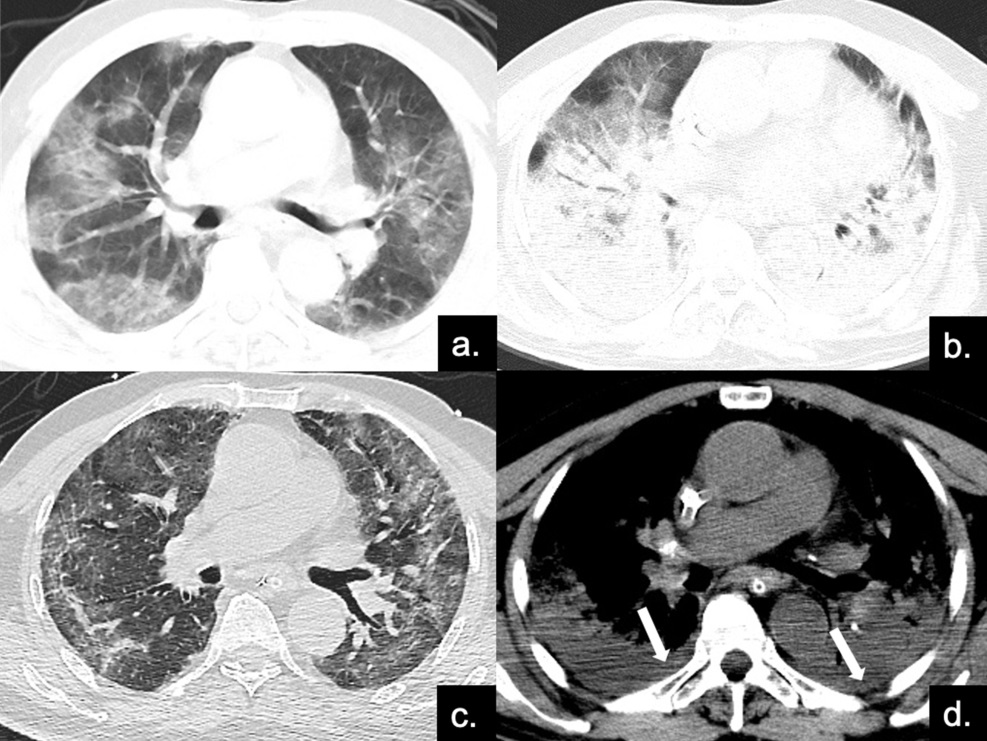
Series CT scans in an 85-year-old man with COVID-19 pneumonia in the severe group. (**a**) Scan obtained on disease day 4, showing patchy ground-glass opacities plus interlobular septal thickening (crazy paving). The Predominant CT pattern was of GGO type, with 12 as total CT score. (**b, d**) Scan obtained on day 10, the disease progressed rapidly to ARDS (PaO2 / FiO2 continued to decrease with a minimum of 85), and the patient was treated with ECMO. Figure b is the lung window, showing diffuse distribution of consolidation and bronchial meteorology in both lungs, mainly dorsal, while the crazy paving can also be seen on the ventral side. Figure d depicts the soft tissue window, and pleural effusion can be found under the dorsal pleura (red arrows). The predominant CT pattern was of consolidation type, with 25 as total CT score. (**c**) Scan obtained on day 40, lung lesions were further absorbed and reticular involvement was increased, accompanied by fibrotic changes. The predominantly CT sign was the reticular pattern, with 17 as total CT score.

In both non-severe and severe groups, the GGO pattern was dominant in the first week, with the severe group showing a higher proportion compared with non-severe cases (18 [72%] and 20 [65%], respectively). Thereafter, the GGO pattern began to decrease substantially, and almost no GGO pattern was observed in the middle and late stages of the disease course in severe cases. In the non-severe and severe groups, the consolidation pattern peaked in the second week, with 9 (32%) and 19 (73%) cases, respectively, indicating an apparently higher proportion in the severe group compared with non-severe cases. Later, the consolidation pattern gradually decreased, and was almost no detectable in both groups after 4 weeks. In the non-severe and severe groups, the reticular pattern began in the second and third weeks, respectively, notably increased thereafter, and became dominant from 4 weeks (both over 40%, **Figs. 2A, 3 & 4**).

### Positive CT features

In the first week of disease onset in the non-severe group, pure GGO accounted for the highest proportion (22/31, 71%), while in the severe group, GGO plus interlobular septal thickening was the most common type (21/25, 84%). In the non-severe and severe groups, the most common imaging sign in the second week of disease was consolidation, with 17 (61%) and 19 (73%) cases, respectively. In the severe group, pure GGO, GGO plus interlobular septal thickening, and GGO plus irregular line showed low proportions after 3 weeks, consistent with markedly decreased single GGO CT scores in the severe group after 3 weeks. In the non-severe group, reticular and fibrotic changes, and GGO plus irregular line were the most common signs from 4 weeks of disease onset, while reticular and fibrotic changes and consolidation were the most common in the severe group (**Table 3; Figs. 2B & 4**).

## Discussion

This study systemically described serial CT findings between non-severe and severe COVID-19 pneumonia cases. According to the above results, both non-severe and severe groups reached the disease peak in the second week after initial symptom onset, and disease scores were higher in the severe group. With disease development, imaging scores in the non-severe and severe groups declined to various degrees, but did not reach 0 point, revealing the presence of massive residual lesions after conversion to a negative nucleic acid test. In the non-severe group, the residual lesions were dominated by GGO and reticular types, while those in the severe group were mainly of consolidation and reticular types. Moreover, we also compared development patterns of non-severe and severe cases at different longitudinal time periods. To the best of our knowledge, this was the first comparative description of a longitudinal thin-section CT series between non-severe and severe patients with clinically and laboratory-confirmed COVID-19.

In the non-severe patient cohort, the disease peak was reached in the second week, corroborating previous findings 11, 12, 23. Meanwhile, we also discovered that many lung lobes were involved in the third week, and maximal diffusion was attained at this moment (in 88% of cases). In the severe patient cohort, disease symptoms peaked in the second week, rapidly progressing from the first week to the second, similar to the development pattern of SARS 23. The peaks in the non-severe and severe groups gradually declined thereafter, but still remained at high levels, which was consistent with previous literature 12, 23. In addition, the SARS follow-up literature also suggested that among 24 patients discharged, 20 show residual lung lesions on chest X-ray films.

We next carried out a semi-quantitative analysis of different lesions, and examined the development trend in this longitudinal research, which has not been mentioned in previous studies. In the non-severe group, GGO remained in a high proportion, and residual lesions at late disease stage were mostly of GGO and reticular type. According to previous findings, GGO is reversible to a large extent 23, 28. Therefore, we speculated that GGO occurrence at the late stage was a sign of disease improvement in non-severe cases, consistent with conclusions drawn from SARS. On the contrary, we demonstrated that GGO proportion was low at the middle and late stages in severe cases, with residual lesions mainly of reticular and consolidation types. A SARS follow-up research 29 indicated that elderly patients in the intensive care unit (ICU) are more likely to develop irreversible pulmonary fibrosis. In this study, median age was higher in the severe group compared with non-severe cases. Consequently, we speculated that severe cases are more likely to develop irreversible pulmonary fibrosis, which should be verified in future studies.

In the first week after initial symptom onset, CT imaging signs in non-severe and severe cases were dominated by the GGO pattern, non-severe cases mainly showed the GGO pattern, while the severe group mostly exhibited GGO accompanied by interlobular septal thickening. Typically, such imaging finding of GGO with or without interlobular septal thickening was similar to previously reported CT features of viral pneumonia induced by SARS and MERS 23, 30-32. However, almost no GGO pattern was observed in the middle and late disease stages in severe cases. These studies also suggested that pure GGO, GGO plus interlobular septal thickening, and GGO plus irregular line account for less than 5% of all positive CT imaging signs, which might be related to more severe disease in the severe group. In the non-severe and severe groups, the consolidation pattern peaked in the second week, with 9 (32%) and 19 (73%) cases, respectively, indicating an overly higher proportion in the severe group compared with non-severe cases. Meanwhile, disease symptoms in the severe group peaked in the second week, revealing that the disease peak is strictly related to the consolidation pattern on lung CT images. At the late disease stage, the reticular pattern was dominant in the non-severe and severe groups; of all positive imaging signs, reticular and fibrotic changes had the highest proportions. The occurrence of reticular changes is usually accompanied by bronchiectasis and structural distortion 23, revealing the possibility of further development into pulmonary fibrosis 29.

This study had some limitations. Firstly, this was a retrospective multicenter study and the time of CT examination was uneven, which may bias CT feature description. Secondly, no pathological analysis was performed in the present study, making it impossible to evaluate the associations of CT features with pathological changes. Finally, a larger sample and longer follow-up are needed to better describe the development of this illness.

In conclusion, COVID-19 in the non-severe and severe groups peak in the second week, and massive residual lesions are found at the late disease stage. In the non-severe group, the residual lesions are dominated by the GGO and reticular type, while those of the severe group are mainly of reticular and consolidation type. The transiently distinct CT manifestations of severe and non-severe cases follow certain patterns at different time points of the disease course, which is related to disease severity, progression, and recovery.

## Figures and Tables

**Table 1:** CT imaging features of patients with COVID-19

CT Characteristics	Definition
Lobe involvement	Categorized as right upper, right middle, right lower, left upper and left lower lobe involvement
The lung segment involved	Each lung segment was reviewed for GGO, consolidation and reticular types, respectively
Lung involvement	Categorized as unilateral or bilateral lung involvement
Lesion location	Central, lesion located in the interior two-thirds of the lung; peripheral, located in the outer one-third of the lung; Both central and peripheral.
Extent of lesion involvement	Categorized as focal, multifocal, diffuse
Predominantly distribution of opacities	Septal/sub-pleural, involving mainly the sub-peripheral one-third of the lung; peri-bronchovasular, surrounding mainly the peri-bronchovascular bundle; random, without predilection for sub-pleural or peri-bronchovascular regions.
Predominantly CT pattern	GGO pattern, an area of hazy increased lung opacity, within which margins of pulmonary vessels may be indistinct; consolidation pattern, a homogeneous increase in pulmonary parenchymal attenuation that obscures the margins of vessels and airway walls; reticular pattern, a descriptive term usually associated with interstitial lung diseases; mixed pattern, combination of GGO, consolidation, and reticulation.
Interlobular septal thickening	Smooth or irregular
Pleural effusion	Fluid in the pleural cavity
Lymphadenopathy	Arbitrary thresholds for the upper limit of normal of 1 cm in short-axis diameters of mediastinal nodes.

**Table 2:** Clinical characteristics of patients with COVID-19

Characteristics	All patients (n=73)	Non-severe (n=47)	Severe (n=26)
Age, year	51±13	49±12	55±15
**Sex**			
Men	43 (59%)	32 (68%)	11 (42%)
Women	30 (41%)	15 (32%)	15 (58%)
**Any comorbidity**	41 (56%)	24 (51%)	17 (65%)
Diabetes	8 (11%)	4 (9%)	4 (15%)
Hypertension	21 (29%)	13 (28%)	8 (31%)
Cardiovascular disease	7 (10%)	5 (11%)	2 (8%)
COPD	3 (4%)	2 (4%)	1 (4%)
Tuberculosis	3 (4%)	3 (6%)	0
Fatty liver	13 (18%)	11 (23%)	2 (8%)
Hepatitis B	10 (14%)	6 (13%)	4 (15%)
**Signs and symptoms**			
Fever	64 (86%)	41 (87%)	23 (88%)
**Highest temperature, °C**			
<37.3	9 (12%)	6 (13%)	3 (12%)
37.3-38	13 (18%)	10 (21%)	3 (12%)
38.1-39	38 (52%)	23 (49%)	15 (58%)
>39	13 (18%)	8 (17%)	5 (19%)
Cough	45 (62%)	26 (55%)	19 (73%)
Chest tightness	41 (56%)	21 (54%)	20 (77%)
Diarrhea	13 (18%)	12 (26%)	1 (4%)
Anorexia	28 (38%)	27 (58%)	1 (4%)
Headache	5 (7%)	1 (2%)	4 (15%)
Myalgia and fatigue	27 (37%)	18 (38%)	9 (35%)
Time of hospitalization	24 (12)	21 (8)	32 (10)
Time from symptoms to image peak	11 (3)	12 (10)	11 (3)
Time from symptoms to PCR positive test	7 (7)	9 (7)	3 (4)
Time from symptoms to PCR negative test	33 (11)	29 (12)	37 (10)

Age is mean±SD; time of Hospitalization, time from symptoms to image peak, time from symptoms to PCR positive test, and time from symptoms to PCR negative test were presented as median (IQR); the remaining data were presented as n (n/N%), where N is the total number of patients with available data.

**Table 3:** CT features of patients with COVID-19

Characteristics	Non-severe					Severe				
	1 week (n=31)	2 weeks (n=28)	3 weeks (n=25)	4 weeks (n=23)	>4 weeks (n=17)	1 week (n=25)	2 weeks (n=26)	3 weeks (n=21)	4 weeks (n=22)	>4 weeks (n=13)
**Lobe**										
Left upper lobe	23 (74%)	20 (71%)	23 (92%)	18 (78%)	15 (88%)	24 (96%)	25 (96%)	21 (100%)	22 (100%)	12 (92%)
Left lower lobe	26 (84%)	26 (93%)	24 (96%)	21 (91%)	17 (100%)	24 (96%)	26 (100%)	21 (100%)	22 (100%)	13 (100%)
Right upper lobe	20 (66%)	20 (71%)	22 (88%)	19 (83%)	15 (88%)	24 (96%)	26 (100%)	21 (100%)	22 (100%)	13 (100%)
Right middle lobe	15 (48%)	18 (64%)	19 (76%)	14 (61%)	11 (65%)	25 (100%)	26 (100%)	21 (100%)	22 (100%)	13 (100%)
Right lower lobe	29 (94%)	25 (89%)	24 (96%)	21 (91%)	17 (100%)	24 (96%)	26 (100%)	21 (100%)	22 (100%)	13 (100%)
**Lung involvement**										
Unilateral	4 (13%)	4 (14%)	1 (4%)	3 (13%)	0	0	0	0	0	0
Bilateral	27 (87%)	24 (86%)	24 (96%)	20 (87%)	17 (100%)	25 (100%)	26 (100%)	21 (100%)	22 (100%)	13 (100%)
**Lesion location**										
Central	0	0	0	0	0	2 (8%)	0	0	0	0
Peripheral	10 (32%)	7 (25%)	6 (24%)	5 (22%)	3 (18%)	4 (16%)	1 (4%)	0	1 (5%)	1 (8%)
Both central and peripheral	21 (68%)	21 (75%)	19 (76%)	17 (74%)	14 (82%)	19 (76%)	25 (96%)	21 (100%)	21 (95%)	12 (92%)
**Extent of lesion involvement**										
Focal	3 (10%)	2 (7%)	1 (4%)	3 (13%)	1 (6%)	0	0	0	0	0
Multifocal	12 (39%)	4 (14%)	2 (8%)	2 (9%)	3 (18%)	11 (44%)	2 (8%)	4 (19%)	5 (23%)	5 (38%)
Diffuse	16 (52%)	22 (79%)	22 (88%)	18 (78%)	13 (76%)	14 (56%)	24 (86%)	17 (81%)	17 (77%)	8 (62%)
**Predominant distribution of opacities**										
Septal/sub-pleural	9 (29%)	7 (25%)	6 (24%)	6 (26%)	3 (18%)	2 (8%)	1 (4%)	1 (5%)	0	0
Peri-bronchovascular	8 (26%)	2 (7%)	0	2 (9%)	3 (18%)	10 (40%)	6 (23%)	3 (14%)	10 (45%)	4 (31%)
Random	14 (45%)	19 (68%)	19 (76%)	15 (65%)	11 (65%)	13 (52%)	18 (69%)	17 (81%)	12 (55%)	9 (69%)
**Predominant CT pattern**										
Ground glass opacity pattern	20 (65%)	8 (29%)	6 (24%)	7 (30%)	4 (24%)	18 (72%)	4 (15%)	0	0	2 (15%)
Consolidation pattern	6 (19%)	9 (32%)	5 (20%)	1 (4%)	0	3 (12%)	19 (73%)	7 (33%)	4 (18%)	0
Reticular pattern	0	2 (7%)	5 (20%)	12 (53%)	12 (70%)	0	0	3 (14%)	11 (50%)	6 (46%)
Mixed pattern	5 (16%)	9 (32%)	9 (36%)	3 (13%)	1 (6%)	4 (16%)	3 (12%)	11 (53%)	7 (32%)	5 (39%)
**Ground glass opacity**										
Number of involved segments	5 (7)	5 (9)	8 (7)	6 (8)	7 (12)	15 (4)	7 (6)	5 (4)	3 (4)	2 (4)
Single ground glass opacity CT score	5 (5)	5.5 (7.3)	6.5 (6)	7 (7)	7 (5)	12 (7)	6 (3)	3 (2)	1 (1)	1 (2)
**Consolidation**										
Number of involved segments	2 (6)	4 (5)	1 (7)	0 (1)	0 (0)	4 (4)	9 (6)	7 (5)	5 (6)	6 (5)
Single consolidation CT score	2 (5)	3.5 (6)	2 (5.8)	0 (1)	0 (0)	3 (2)	13.5 (6.8)	9 (4)	8 (5)	7 (6)
**Reticular**										
Number of involved segments	0 (0)	0 (1)	1 (5)	3 (7)	4 (7)	0	1 (5)	5 (6)	7 (8)	8 (5)
Single reticular CT score	0 (0)	0 (1.3)	1.5 (5)	2 (6)	4 (4)	0 (0)	0 (2)	8 (2)	9 (2.8)	10 (2)
Total CT score	7 (7.5)	9.5 (8.5)	10 (6)	9 (7)	9 (11)	15 (7)	20 (2.8)	20 (4)	19 (6.5)	18 (2)
Pure ground-glass opacity	22 (71%)	13 (46%)	10 (40%)	8 (35%)	7 (41%)	11 (44%)	2 (8%)	1 (5%)	1 (5%)	1 (8%)
Ground-glass opacity plus Interlobular septal thickening	8 (26%)	4 (14%)	1 (4%)	0	0	21 (84%)	14 (54%)	4 (19%)	0	0
Ground-glass opacity plus irregular line	12 (39%)	13 (46%)	16 (64%)	12 (52%)	8 (47%)	3 (12%)	6 (23%)	18 (86%)	8 (36%)	4 (31%)
Reticular	1 (3%)	4 (14%)	11 (44%)	12 (52%)	11 (65%)	0 (0%)	1 (4%)	13 (62%)	22 (100%)	12 (92%)
Fibrotic changes	3 (10%)	12 (43%)	16 (64%)	13 (57%)	14 (82%)	1 (4%)	2 (8%)	12 (57%)	21 (95%)	13 (100%)
Consolidation	18 (58%)	17 (61%)	14 (56%)	5 (22%)	4 (24%)	4 (16%)	19 (73%)	17 (81%)	20 (91%)	8 (62%)
Traction bronchiectasis	0	0	1 (4%)	2 (9%)	2 (12%)	2 (8%)	4 (15%)	3 (14%)	9 (41%)	7 (54%)
Pleural effusion	0	0	0	0	0	2 (8%)	4 (15%)	3 (14%)	1 (5%)	0

The number of involved segments, and single ground glass opacity, single consolidation, single reticular and total CT scores were presented as median (IQR), the remaining data were presented as n (n/N%), where N is the total number of patients with available data.

## Abbreviations

COVID-19: Corona virus disease 2019
SARS-CoV-2: Severe acute respiratory syndrome coronavirus 2
SARS: Severe acute respiratory syndrome
MERS: Middle East respiratory syndrome
GGO: Ground-glass opacity
